# Psychological Mechanisms Involved in Radicalization and Extremism. A Rational Emotive Behavioral Conceptualization

**DOI:** 10.3389/fpsyg.2019.00437

**Published:** 2019-03-06

**Authors:** Simona Trip, Carmen Hortensia Bora, Mihai Marian, Angelica Halmajan, Marius Ioan Drugas

**Affiliations:** Department of Psychology, University of Oradea, Oradea, Romania

**Keywords:** radicalization, extremism, irrational beliefs, psychological mechanisms, absolutistic demands for fairness, uncertainty intolerance, global evaluation of human worth

## Abstract

Extremist acts and the process of radicalizations got into researchers’ attention worldwide since 2001. The aim of this paper is to offer a broad image on radicalization and extremist acts and to bring a new perspective for the conceptualization of radicalization. Radicalization is a process of developing extremist beliefs, emotions, and behaviors. The extremist beliefs are profound convictions opposesd to the fundamental values of society, the laws of democracy and the universal human rights, advocating the supremacy of a certain group (racial, religious, political, economic, social etc.). The extremist emotions and behaviors may be expressed both in non-violent pressure and coercion and in actions that deviate from the norm and show contempt for life, freedom, and human rights. A complete inroad to psychological mechanism involved in the process of radicalization is offered in order to have a broad image regarding current research in the field. Starting from this point, a rational emotive and behavioral conceptualization on radicalization has been developed, bringing together all the concepts and knowledge in the field. A complete and clear conceptualization is crucial for developing prevention/intervention programs and good practices in dealing with this process which has been spreading in the past years. The final part deals with directions regarding prevention/intervention programs from a rational emotive and behavioral perspective, and also from the perspective of European policies.

## Psychological Mechanisms Involved in Radicalization and Extremism. A Rational Emotive Behavioral Conceptualization

Since the attacks on September 11^th^, 2001, significant efforts have been made in order to explore and better understand radicalized behaviors, especially terrorist acts. The term radicalization was brought into the research interest after the 2005 terrorist bombing attacks that targeted the public transportation in London, both on ground and underground.

### Definition of Terms

Many authors have offered definitions of radicalization. McCauley and Moskalenko (2008) present both a functional and a descriptive definition of radicalization. From a functional point of view, radicalization is defined as an enhanced preparation for intergroup conflict and an accentuated engagement to it. From a descriptive point of view, radicalization refers to a change in beliefs, feelings, and behaviors that justify intergroup violence and the demand for sacrifice in defending the own group. Bott et al. (2009) assumed the U.S. Department of Homeland Security’s definition of radicalization as embracing extremist beliefs that support violence as a method to effect societal change. Radicalization is a process by which people develop extremist ideologies and beliefs (Borum, 2011). Schmid (2013) conceptualized radicalization as being both an individual and group process, whereby political actors and groups that are politically polarized renounce dialog, agreement, and tolerance and use either non-violent pressure and coercion, or various forms of political violence including violent extremism (terrorism and war crimes).

How extremism is defined in literature? Extremism refers both to political ideologies and to methods through which political actors try to achieve their aims. Extremist political ideologies oppose the fundamental values of society and the principles of democracy and universal human rights by advocating racial, political, social, economic, and religious supremacy. The methods show disregard for others’ life, liberty, and human rights (Neuman, 2010). The extremist beliefs are zeal or profound convictions and the extremist behaviors are reactions that deviate from the norm (Klein and Kruglanski, 2013).

To summarize, radicalization is a process of developing extremist beliefs, emotions, and behaviors. The extremist beliefs are profound convictions that oppose the fundamental values of society, the laws of democracy and universal human rights by advocating the supremacy of a particular group (racial, religious, political, economic, social etc.). The extremist emotions and behaviors may be expressed both in non-violent pressure and coercion and in actions that deviate from the norm and show contempt for life, freedom, and human rights.

There is a clear distinction between terms counter-radicalization, de-radicalization, and disengagement. Counter-radicalization involves social, political, legal, and educational prevention programs designed to discourage disgruntled and perhaps already radicalized people from becoming terrorists (United Nations, 2008). De-radicalization and disengagement refer more to the intervention programs. De-radicalization implies a cognitive trajectory, thus programs focus on changing the cognitive framework of radicalized individuals with the aim of discouraging their involvement in violence and re-integrating them into society (United Nations, 2008; Schmid, 2013). Disengagement implies changes in behaviors by abandoning the association with violent groups and by not using violence (Butt and Tuck, 2012).

### An Exploration of Psychological Mechanisms Involved in Radicalization and Extremism

Following the Prevent Pyramid model, most already existing counter-radicalization, de-radicalization, and disengagement programs are targeted, interventionist, and enforcement approaches. The lowest level of the pyramid represents all the members of a community, the second targets the most vulnerable of these, the third level expresses the line that will be crossed by some of the more vulnerable members to move toward radicalization, and the fourth level focuses on individuals that are actively breaking the law (Audit Commission, 2008; McCauley and Moskalenko, 2008; Young et al., 2013). Can we do something more? If our efforts would be focusing on the base of the pyramid, what would be the chances to decrease the number of people who cross into the next levels?

Demant et al. (2008) concluded that in general, disengagement in radicalized behavior is linked with the de-radicalization of beliefs. Their qualitative results supported that changes in behaviors and in beliefs do not always go hand in hand; sometimes radical behavior can be stopped without a moderation of radical beliefs, whereas some individuals may have radical beliefs but are not members of a radical movement, and do not carry out violent actions. Usually, when research papers describe radicalized beliefs, they make referral to radicalized ideology. But what basic psychological mechanisms put people in the vulnerable position of believing in radicalized ideology? Which are the basic psychological mechanisms preventing people from believing in radicalized ideology?

Wiktorowicz (2005) introduced the notion of “cognitive opening”—the moment when an individual who faces discrimination, socioeconomic crisis, and political repression is trying to understand life events and suddenly his previously accepted beliefs are shaking and he becomes vulnerable and receptive to the new way of thinking—radicalized ideology. What are the cognitive factors that facilitate this cognitive opening?

Moghaddam (2005) talks about the perception of the elements of unfairness and injustice. The individual thinks that his group does not have the same advantages as other groups, beliefs that sometimes are not supported by empirical evidences. Low levels of educational and socioeconomic backgrounds were not found to be characteristic of terrorists (Krueger and Maleckova, 2003). These absolutistic demands for fairness are the starting point of the cognitive openness to radicalization. The rigid us versus them, good and evil categorical thinking lead to displaced aggression directed to other target (Western countries) which is not the trigger of frustration and then to justification of terrorist acts through desire to achieve an ideal society.

In line with Moghaddam’s results, Doosje et al. (2013) found the extent to which people experience deprivation both as individual and as member of a group predict the radical belief system’s determinants. One of these determinants is perceived injustice, which in this model predicts perceived societal disconnectedness, defined as a perception that individual does not belong to the mainstream of the society, an idea that feeds violent attitudes.

Doosje et al. (2013) found more paths of developing radical belief system and violence. One starts with collective deprivation, continues with symbolic threats, in-group superiority and attitude toward violence. Another path includes realistic threats activated by both individual and collective deprivation that is a predictor of perceived distance toward other people that leads to violent attitude. In the following, we will analyze each path separately.

According to integrated threat theory (Stephan and Stephan, 2000), members of an in-group expect out-group members to behave in ways that are detrimental to in-group members. When an individual faces deprivation as a group member, he may believe that his group’s morals, values, standards, beliefs, and attitudes are more correct. This difference between in-group and out-group is a symbolic threat that can lead to cognitive evaluation of in-group as being superior to out-groups, belief that supports violent attitudes. In situations in which someone may experience deprivation either as individual or as a member of a group, he/she may expect out-group members to behave in ways that are a threat to the very existence of the in-group (realistic threat), create a great distance toward people of the out-group, and develop violent attitudes. So, can these expectations be another starting point for cognitive openness to radicalization?

Another path starts with perceiving collective deprivation that activates emotional uncertainty which, in turn, is a predictor for in-group superiority (the members of in-group consider themselves to be superior to all other groups). Perceived in-group superiority was the best predictor of attitude toward violence. The authors conceptualized personal uncertainty as feelings, defining it as a subjective sense of doubt in self-views, world-views, or in relationship between them. By analyzing the scale used by authors, it can be concluded that emotional uncertainty was measured as an emotion experienced by an individual when facing uncertainty in his life. Thus, life events in which an individual faces deprivation as group member may be perceived as uncertain and activate feelings like anxiety, depression, or anger. This is a moment of maximum vulnerability when radical ideology offers a solution for this personal uncertainty by introducing meaning and a focus of life. The emotional distress experienced in a situation evaluated as uncertain leads to cognitive evaluation of in-group as being superior to out-groups, belief that supports violent attitudes. Is there any cognitive mechanism that opens the door for emotional distress when we face uncertainty in life?

Uncertainty-identity theory (Hogg and Adelman, 2013; Hogg and Wagoner, 2017) postulates that people are motivated to reduce self-uncertainty, specifically feelings of uncertainty about their life, their future, and uncertainty about their self and identity. One way to solve this problem of self-uncertainty is group identification. Individuals use the groups that they are part of to define their self-concept (social identity theory, Tajfel and Turner, 1979). Social groups are represented as prototypes, sets of attributes, values, beliefs, feelings, behaviors that define the group and its members and distinguish it from other (self-categorization theory, Turner et al., 1987). Therefore, by prescribing prototypes, groups provide people identity and reduce uncertainty regarding who they are, how to behave, and what to think, and who others are and how they might behave, think. When self-uncertainty becomes chronic, pervasive, or acute, people are strongly attracted to extremist groups, because they prescribe a clear prototype for how one should behave, think, and feel in all situations, and how to behave toward out-group members (Hogg and Wagoner, 2017). Self-uncertainty drives people toward distinct and clear groups, motivates them to defend their in-group against out-groups who are perceived as threat for their group’s values and beliefs. What exactly is self-uncertainty, cognition, or emotion?

Lüders et al. (2016) redefined self-uncertainty as self-concept uncertainty and conceptualized it as epistemic vagueness, the need for meaning and epistemic equilibrium. Anxiety-to-approach model of threat and defense (Jonas et al., 2014) postulates that when people face different threats of self, they experience raised anxious uncertainty as a consequence of behavioral inhibition system activity and they try to escape from it by using reactive defensive strategies. Authors include in the threats of self category, three types of cognitions: (1) the need for meaning and epistemic understanding is not met; (2) self-esteem of the individual is devalued by others, so he/she does not meet the expectations of his/her group members; and (3) threatened self-control over their action, physical, and social environment.

Reactive approach motivation theory (McGregor et al., 2013) considers motivational conflict as the base of personal uncertainty that is conceptualized as anxiety. An individual feels anxiety when he is impeded or cannot reach his goals. Both models postulated that some people show persistent behavioral inhibition system activity and as a consequence they experience decreased life satisfaction, increased state of anxiety and social avoidance. Most people try to manage the threat in order to decrease anxiety, and engage in defensive compensatory acts/approach-oriented distal defenses.

McGregor et al. (2013) concluded that extreme religious beliefs were determined by personal uncertainty enabled through defeating an active achievement goal. Extreme conviction and idealistic approach were used as defense when individual feels uncertain and faces personal uncertainty through active goal threatening (achievement, relationship). Insecure forms of high self-esteem (high explicit, but low implicit) were associated with defensive compensatory conviction. McGregor et al. (2005) empirically support the idea that high self-esteem can be a sign of defensiveness and may result from repeatedly hiding implicit self-doubts with the display of explicit self-worth masks (pride, avoidant arrogant attachment style, narcissism). People with secure high self-esteem (high explicit and high implicit self-esteem) do not take things personally when they are confronted by others. Compensatory control theory (Kay and Eibach, 2013) stipulated that people wish to live in a controllable, predictable world, but because our world is one of chance and uncertainty, they try to decrease their anxiety through the beliefs of personal, governmental, or religious control. Groups represent a fruitful source for approach-oriented defense mechanisms that help people maintain their need for epistemic equilibrium, self-esteem, belonging, and control.

According to goal systems theory, extremism is an expression of goal commitment, the zeal represents a direct expression of commitment to a focal goal and deviant behaviors are indirect expression of this (Klein and Kruglanski, 2013). Research showed that high commitment to a valued, focal goal conducts to alternative goals’ suppression (Kopetz et al., 2011). What is the focal aim of the extremist people? Can we replace it with a similar goal, highly feasible with the focal goal?

In the following section, we will try to offer an answer to all the questions raised above by conceptual relationship between all the theories mentioned above and the philosophy of rational emotive behavioral therapy.

### A Conceptualization of Radicalization From Rational Emotive Behavioral Psychotherapy’s Perspective

Rational emotive behavioral psychotherapy (REBT) can help radicalization and extremism prevention programs to have a cleared framework and to get more efficient. One first argument is brought by Harrington (2013) who affirms that extremist leaders have the ability to address the irrational part of human beings. As well as the theories mentioned above, REBT postulates three main intertwined aspects of human function: beliefs, feelings, and behaviors. One basic principle is that evaluative cognitions are the most important determinants of human emotions and behaviors (Ellis, 1994).

DiGiuseppe et al. (2014) differentiate inferential beliefs from evaluative beliefs. The first category describes peoples’ perceptions of reality and the inferences they make based on what is perceived. The above-mentioned models talk about extremist peoples’ perception of individual and collective deprivation, realistic and symbolic threat. let us suppose that an individual perceives a deprivation of his/her group (employers hire immigrants who can be paid a lower salary) and makes the inference that his/her group’s morals, values, and standards regarding work are more correct then immigrants’ or employers’ (symbolic threat). These cognitions may be true, accurate, in accordance with reality or false, inaccurate, and empirically inconsistent. They are associated with emotional disturbance (anger, anxiety, depression), but REBT recognizes evaluative beliefs as being central to emotional distress and calls them irrational beliefs. These irrational beliefs are almost always unconscious, are logically inconsistent, are not supported by empirical reality, do not help us attain our goals, and are absolutistic and dogmatic.

Ellis (1994) proposed that rigid and absolutistic demands are the core of emotional distress, all other categories of irrational beliefs arise from this dogmatic root. Humans easily transform their preferences, wishes, and desires into must, should, ought, and commands. There are three categories of absolutistic demands: (1) self-demands; (2) other-demands; and (3) world-demands. Ego-oriented demandingness (e.g. I must perform well, I must prove my competence, I have to be competent, I must have control, I must please my friends) leads to self-hate, anxiety, depression, and suicidal behavior. Other, directed demandingness (e.g. others must treat me always nicely, fairly; he/she must love me) leads to strong feelings of anger, rage, hurt, and violent disruptive behavior. World-demandingness (e.g., the world, the environment, the economic, social, political conditions which I live in must be favorable, safe, enjoyable, fair, just, without hassle; there must be certainty in the world) leads to self-pity, anger, depression, anxiety, despair, and to dysfunctional behaviors such as withdrawal, addictions, and violence.

As we have already seen, all models of psychological factors that make people vulnerable to radicalization and extremism mention absolutistic demands for fairness and certainty even though they do not use this concept of demandingness. We think that this concept brings more clarity to these models. Therefore, people facing different life events perceive them as a deprivation or threat, make inferences about them (we are different, our values are better) and endorse demandingness beliefs (these deprivations or threats should not happen to us; what happened was not fair as it always must be; I must be certain that this situation will not be repeated; I must stop it and change it into a just world; others have to behave always fairly). Extremist leaders address this dogmatic, irrational part of humans, they claim for absolute ideals as ideal world should exist and people that ignore this must be re-educated or eliminated. In both sides, that of vulnerable people and that of extremist leaders, there is a confusion between “what is preferred” and “what is demanded” (Harrington, 2013, p. 172).

Ellis (1986), p. 148 offers some examples of extremist demandingness beliefs: “Our views of people and universe are absolutely and everlasting true and nobody deserve to live who opposes these supreme views. Our political or religious cause is the only worthy one that should exists. We can save humanity and prevent evil. We must do anything to make sure that we extirpate everyone who prevents our noble cause from prevailing”. Both at personal level and at the extremist ideology level, freedom and absolute beliefs are incompatible. Analyzing the terrorist attacks on the World Trade Center and the Pentagon that took place on September 11^th^, Ellis (2003) identified the absolutistic demands that terrorist could hold: (1) they absolutely had to punish America (self-demands); (2) Americans must absolutely not oppose their standpoint (other demands); and (3) the world should be fair and just (world-demands).

Two main categories of irrational beliefs are logical derivatives from absolutistic demandingness: low frustration tolerance beliefs (discomfort disturbance beliefs) and global evaluation of human worth (ego disturbance beliefs). Low frustration tolerance (LFT) is a belief stating that reality must be as one wants it to be—easy, effortless, perhaps pleasurable, and comfortable; individuals think that he is not able to withstand aversive internal and external states elicited by an aversive experience. REBT encourages responsible hedonism reached through the ability to inhibit a response to immediate negative reinforcement or reward and to pursue alternative future reinforces that may be available with achieving long-term goals. LFT impedes people from achieving long-term goals, focusing their attention on short-term goals. Dryden (1999) and Harrington (2005) advocate for a LFT multidimensional concept, some of the LFT components being uncertainty intolerance, emotional intolerance (intolerance of emotional distress), entitlement (intolerance of unfairness and frustrated gratification), discomfort intolerance (intolerance of difficulties and hassles), and achievement intolerance (intolerance of frustrated achievement goals). Uncertainty intolerance generates and maintains anxiety disorders, people are more likely to interpret all ambiguous stimuli as threatening and therefore exacerbate their anxiety and engage in avoidant or reassurance-seeking behaviors (Carleton, 2016).

Global evaluation of human worth is a generalized evaluation and denigration of self as well as other. Most of the people practice conditional acceptance of self and others rather than unconditional acceptance. They evaluate themselves and others as being good, valuable human beings when they behave well, when they do a good job, when they have success. When they and others do bad acts or sins, they start to condemn themselves (self-downing) and others (other-downing), thinking that they are not valuable human beings, that they are bad and rotten persons. On the contrary, unconditional self/other acceptance means to understand that all people are imperfect creatures, fallible human beings. There is no perfect human being; all people have qualities and weakness. All people sometimes behave badly and sometimes well, but people are more than their individual behaviors. Global evaluation of human worth is the base for categorical thinking (us-vs.-them), perceived societal disconnectedness, distance toward other people, in-group superiority.

Some of the above-described paths to radicalization and violence are clear. Everything starts with perceived deprivation. There is a path that continues with absolutistic demand for fairness followed by global evaluation of human worth (the in-group and the self are evaluated as being superior, valuable and others are judged as being inferior and evil). It is not very clear if demand for fairness is associated with entitlement (I cannot stand unfairness), but this association is plausible. Another path places global evaluation of human worth (in-group superiority, out-group inferiority; distance toward other people)right after the perception of deprivation.

The concept of personal uncertainty mentioned by the models of extremism is not so clear and it mixes LFT with uncertainty and self-downing beliefs. Reinterpreting the models, we suggest that perception of deprivation activates absolutistic demands for certainty, associated with LFT beliefs (the world I live in must be certain, if not I cannot stand it, I cannot face it) that cause emotional distress (anxiety) which in turn continues the vicious circle, pushing the individual to look for certain stimulus (entitative extremist groups) which he/she then evaluates as superior (global evaluation of human worth). Concepts such as uncertainty identity, epistemic vagueness, low self-esteem, insecure form of high self-esteem are nothing but self-downing (I am nothing, I am a bad person, I am a worthless human being). So, when people face events that block them from reaching their goals, they may develop self-downing beliefs and then embrace compensatory conviction (extremist ideology).

Ellis (2003) mentioned that terrorists involved in September 11^th^ attack first considered themselves worthless and powerless (self-downing) because they are not able to stop America from exporting its economy, industry, culture to the Muslim countries. In order to prove that they are worthwhile and powerful individuals, they have to punish America. Americans are devils because they oppose their standpoints, so Americans deserve to be destroyed. It is impossible to be happy in this evil world, so they must kill the devil Americans and all other worthless people in order to attain the eternal, blessed life. By doing so, they believed they were doing something just. The question of justness was raised by Catharine McLaren, one of REBT experts who examined the impact of the events of September 11^th^ (Weinrach et al., 2004): “Who decides which deaths are just?” All the experts, along with Albert Ellis underlined that global evaluation of others as evil leads to committing atrocities against them and serves as justification for these atrocities. But at the same time, global evaluation of others as being worthless and evil increases the chances that they will consistently behave with this view. As Kristene Doyle and Dominic DiMattia mentioned in that interview, behind the World Trade Center attacks were the rigid, dogmatic convictions along with condemnation beliefs.

As we could see, some paths show a flow of beliefs from irrational beliefs to extremist mindset or radical belief system, others mention in-between dysfunctional emotions (uncertainty anxiety).

There is no other psychotherapeutic school that makes a more clear distinction between inappropriate, dysfunctional, and appropriate, functional emotions than REBT. Both positive and negative emotions may be adaptive or disturbed. DiGiuseppe et al. (2014) and Walen et al. (1992) described five levels at which dysfunctional emotions can be differentiated from functional ones. Disturbed emotions are activated as long as an individual accesses irrational beliefs, and this cognitive level was already described. At the phenomenological level, dysfunctional emotions lead to the experience of an intense psychic sufferance or discomfort (intense, long lasting anxiety/depression/anger/guilt/shame/hurt). This is the moment of maximum vulnerability to radicalization and extremism (Doosje et al., 2013; McGregor et al., 2013; Jonas et al., 2014; Lüders et al., 2016). At the physiological level, inappropriate emotions express through much stronger nervous system hyperactivity. Behaviorally, they motivate people to engage in self-defeating behaviors and in behaviors that block them to reach their goals or to solve the problems they encounter. At the social level, they push people to engage in behaviors related to insecure attachment (they look for extremist groups).

DiGiuseppe and Tafrate (2010) discriminate between healthy, adaptive anger and disturbed anger. When people face unpleasant life events and perceive them as opportunities for others to take their own resources and status, an undifferentiated emotional state arises which in turn activates two motives: (1) to flee or avoid the negative situation; (2) to control or eliminate the unpleasant stimulus. In the next phase, the appraisal processes enters the scene. The individual appraises the strength of the threat and its resources to deal with it, these appraisals can lead to fear, anger, and depression. The motive consistent with one of these emotions is further strengthened and the inconsistent motive is eliminated. When the appraisal activates anger, the motive of control or attack is strengthened and the motive of flee is dissipated. As the level of anger increases, the urge of the motive will increase. Disturbed anger includes the motive to harm others again and again, more than necessary to maintain social order. Two such motives are revenge end envy. The revenge motive is related with concepts of fairness and equity, the envy motive is connected with power and dominance. An angry person will be vulnerable and easily influenced by extremist propaganda that asserts that others, especially Western countries, are cheating, are not respecting the rules they agreed upon resource allocation. In consequence, the person will seek revenge, will want the Western countries to pay or suffer for their behaviors. The terrorist acts are ways to get revenge. The extremist propaganda may also focus on competition and dominance. This will feed the motive of envy, the person believes that power is necessary for his/her survival. He/she can also feel envy for Western countries’ prosperity. The terrorist acts are ways to express the desire to have greater power than the attacked country. Western countries have prosperity here on earth, but she/he will have prosperity in heaven (envy); meanwhile Western countries will burn in the fires of hell (revenge). The terrorist martyrdom may be based on envy, people think they lost or never had a “symbolic” position in the social rank, and the martyrdom gives them that position.

Healthy and adaptive anger generates healthy and adaptive motives of changing the environment. Sometimes aggression is necessary to change the environment. For example, the revolutionaries in the 1989 Anti-communism Revolution in Romania were angry toward Ceausescu’s regime and manifested some aggressive behaviors (breaking things, screaming), but their motive was to control and establish democracy. Quoting Averill (1993), the above-mentioned authors characterized functional anger as targeting corrective actions, being directed only to the responsible persons not to the innocent third party, preventing the occurrence of an attack or offence, being proportional with the offence and involving problem solving. None of these characteristics are specific to anger that could lead to extremism.

What is very specific to REBT is the idea that people make irrational appraisals not only regarding life events, but also their disturbed feelings and behaviors. In what concerns emotions associated with radicalized act, an individual may think: I should not feel like this; I cannot face my sufferance; what a worthless person I am; I am entitled to be angry, I am right. Here are some examples of thoughts regarding extremist behaviors: I did what I had to do; I am so good, I want to change this world. Extremism is the perfect solution to our society’s problems. All justifications brought by extremists for their acts are based on irrational beliefs they have about their acts.

The secondary appraisal may explain why changing extremist beliefs and extremist behaviors do not always go hand in hand. Theories of planned behavior (Ajzen, 1985) postulated that attitudes toward behavior, the individual’s subjective norms regarding behavior, and the perceived control over behavior mediate changings in behaviors. According to goal systems theory, the beliefs that extremists could have about their behavior (secondary appraisal) may prime their extremist valued goal that in consequence suppresses alternative goals and perpetuates individual’s violent behaviors.

How can this conceptual clarification help us prevent radicalization?

### Rational Emotive Behavioral Education can Offer an Answer

Moghaddam (2005) argued that the fight against violent extremism is lost if the programs are not focused on the ground floor conditions because if these are not changed, every violent extremist who is eliminated can be rapidly replaced by others. What better way than education to reach people from the ground floor of the preventive pyramid? let us not forget that the development of socially and morally responsible citizens has always been an educational ideal.

Council of Europe stipulates that in order to participate in a culture of democracy, citizens need to learn and practice four democratic competences: (1) values (valuing human dignity and human rights, valuing cultural diversity and valuing democracy, justice, fairness, equality and the rule of law); (2) attitudes (openness to cultural otherness, respect, civic-mindedness, moral responsibility, self-efficacy and tolerance to ambiguity); (3) skills (autonomous learning skills; analytical and critical thinking skills; skills of listening and observing; empathy; flexibility and adaptability; linguistic, communicative and plurilingual skills; co-operation skills and conflict-resolution skills); and (4) knowledge and critical thinking (politics, law, human rights, religions, history, economies, environment, and cultures) (Barrett and Council of Europe 2016).

In order to minimize absolutistic thinking and make progress toward mental health, Ellis (1986) advocated the worldwide use of scientific counseling and psychotherapy as being incorporated into large-scale education initiatives in schools, community, churches, mass-media. One of such possible initiatives is rational emotive behavioral education (REBE). REBE is an extension of REBT in education, and it is focused on prevention by teaching mental health skills to students, teachers, and parents in order to efficiently face life events. REBE can complete education for democracy through different kinds of strategies that can be used: class activities, activities with small groups, exercises into major subjects teaching classes, extra-curricular activities, recreational play, etc.

Trip et al. (2007) showed that REBE had a powerful effect on decreasing dysfunctional behaviors and irrational beliefs and a moderate effect on changing inferential beliefs and dysfunctional emotions. More recent meta-analysis (David et al., 2017) pointed out that changes in irrational and rational beliefs is highly associated with changes in outcomes (emotions, behaviors, other cognitions, quality of life, school performance, social skills, physiological, and health outcomes). Their results support REBT as an efficient intervention, showing different effect sizes in modifying and maintaining cognitive (other cognitions), emotional, and behavioral outcomes, in both between and within situations.

Unconditional self and other acceptance is fundamental for valuing human dignity and human rights. If the belief that all human beings are of equal worth becomes a guiding principle in people’s life, then people will behave with respect, compassion, will defend fundamental freedom, and will protect human rights. Knowing that there is no perfect human being, individuals will appreciate and learn from the pluralism of opinions and practices offered by our world’s cultural diversity. Unconditional self and other acceptance helps people to take into consideration perspectives of other people, use dialog and co-operation. Openness to other people involves willingness to give up judgment of other people, what is in fact unconditional other acceptance. It also helps interrelationships, thus people will engage more in activities with different others and they will be more able to offer empathy. Respect means positive regard and esteem for others, which is unconditional other acceptance. Those who know their qualities and weaknesses take the responsibility more easily for both positive acts and misbehaviors. Unconditional self and other acceptance increases the sense of belonging. REBT accepts one inelegant solution to global evaluation: that all humans are worthwhile because they are alive, but encourages us to practice the one elegant view—humans are neither worthwhile nor worthless since they are too complex to be globally rated; “It is impossible to rate my whole person as ‘good’ or ‘bad’ because I am an exceptionally complicated and ever-changing individual. So I shall do my best to only rate my behaviors as ‘bad’ when they sabotage my own and my community’s basic goals, purposes, and values” (Ellis, 2006, p. 291).

Teaching and practicing unconditional self-acceptance help people face negative life events and to not develop self-uncertainty: (1) they know who they are, they recognize their mistakes and limits and use their strengths in order to correct the mistaken behaviors; they will know how to differentiate their own person from their behaviors; they will change the discourse of “I do not know how I became like this, I don’t know who am I anymore, I am pathetic and worthless, I do nothing and I let my people suffering” with “I know who am I, I know what are my strengths and my limits. I know how to use them in order to me and my group have a better life”; (2) they will be able not to equate others’ expectations toward them with their own value as human being; they will change the message of “I am no good, if I do not meet the expectations of my group or I am the best in the world because I did what other expect me to do” with “I know my limits, I recognize my mistakes, I know my group expected from me to behave in a right way, but I am fallible human being. I can use my strengths to correct my mistakes. Even know they blame me for my behaviors, I know I am not what I am doing. I did a good job, I did what other expected from me, but it doesn’t mean I am a perfect creature, I know my strengths and my limits”; (3) they accept the fact that there is no good or bad people but only people that are doing good and bad acts, they will not invest a lot of control in their action and their environment in order to show that they are somebody else.

Practicing unconditional other acceptance, people accept that sometimes people do exceptionally bad things, as the terrorist and suicide bombers do, but still they are not what they do. “They are what they do” is an overgeneralization (Ellis, 2006). In this article, we evaluate their ineffective thoughts, behaviors, and emotions, but we did not rate them globally as being evil, bad, or worthless. Unconditional other acceptance means to accept the sinner but not the sin. This helps us to practice forgiveness. Religion preaches forgiveness and not condemning the self. Jesus Christ is a model of unconditional other acceptance and forgiveness. Allah is forgiving and merciful. Unconditional other acceptance and forgiveness will impede people from responding to an evil deed with an evil deed, but rather solve the problem.

REBE can help people to evaluate life situations in a rational way in terms of tolerance to uncertainty beliefs (I can face, I can deal with uncertain and ambiguous situations) leading them to adaptive inferential beliefs (ambiguous stimuli perceived less threatening), less anxiety, and more approaching behaviors. Accepting the fact that we all live in a world of probability and chance and there is seldom gain without pain can make a difference in people’s engagement in day-to-day or civic tasks, by assuming risks and responsibilities and also by decreasing their anxiety (Bora et al., 2018a,b). Rational thoughts like “I’d like that all decisions taken in this world to be fair and just, I can face the unjust decisions, I know this is impossible because people are fallible” help people to find peaceful ways of civic engagement with political decision. Developing rules tolerance and decreasing entitlement beliefs (I deserve more goods, services, or special treatment than others and it is not fair when this does not happen) create a sense of social justice and responsibility.

REBE can teach people the distinction between the preferences or desires and the demands, to challenge their expectancies or demands that their desires must be met and to develop the rational alternative such as: Just because I want others to always behave fair, does not mean that they will do it. Understanding the fact that things do not have to happen just because we wish them leads to problem-solving. A demand that our desires must be met is equal with the demand that the problem should not exist.

By learning and practicing rational beliefs, people could be helped to develop flexibility and adaptability. People will be able to reconsider their opinion in the light of new evidences, to control and regulate their emotions and to adjust their behaviors in a socially appropriate way.

## Author Contributions

All authors listed have made a substantial, direct and intellectual contribution to the work, and approved it for publication.

### Conflict of Interest Statement

The authors declare that the research was conducted in the absence of any commercial or financial relationships that could be construed as a potential conflict of interest.
